# 3D intelligent printing technology-assisted training improves core competencies of clinical medicine interns: bridging undergraduate further education and community health service needs

**DOI:** 10.3389/fpubh.2026.1788437

**Published:** 2026-04-30

**Authors:** Qiongting Luo, Xinyu Wang, Wenwen Hou, Xiaofen Yu, Zheng Wang

**Affiliations:** Hangzhou Medical College (Affiliated People’s Hospital of Hangzhou Medical College, Zhejiang Provincial People’s Hospital), Hangzhou, China

**Keywords:** 3D intelligent printing technology, clinical medicine interns, community employment, core competencies, further education, medical education reform

## Abstract

**Introduction:**

Clinical internship education for Chinese clinical medicine interns faces dual pressures of postgraduate entrance exams and the National Medical Licensing Examination (NMLE), with persistent flaws in self-directed, mobile, and transformational learning. 3D Intelligent Printing Technology (3DIPT) has been widely applied in the surgical system for medical education and surgical planning, yet its value in community medicine-oriented internship training remains unclear.

**Methods:**

This quasi-randomized controlled trial enrolled 120 interns (observation group: 3DIPT-assisted teaching, *n* = 60; control group: traditional teaching, *n* = 60) to evaluate core competency changes.

**Results:**

After 6 months, the observation group showed significantly higher scores in simulated NMLE anatomical imaging tests, Social Medical Curiosity (SMC), self-directed anatomy learning, mobile learning willingness, and transformational learning ability (all *p* < 0.05).

**Discussion:**

3DIPT-assisted teaching may effectively improve interns’ core competencies for community medicine practice, providing a feasible dual-track training strategy for balancing postgraduate education and community physician cultivation in developing countries.

## Introduction

Clinical internship is a critical transitional phase for undergraduate clinical medicine students, integrating theoretical knowledge into clinical practice and laying the foundation for future careers (1). In developing countries represented by China, clinical medicine interns face a unique dilemma: they must simultaneously prepare for postgraduate entrance examinations (to secure optimal academic opportunities) and the NMLE (to qualify for community practice if postgraduate studies are unsuccessful) (2). The NMLE pass rate in China has remained between 43 and 61% in recent years (3–5), reflecting prominent issues in internship education such as weak learning interest, poor application of knowledge to solve clinical problems, insufficient self-directed and mobile learning abilities, and lack of transformational learning (6).

3D Intelligent Printing Technology (3DIPT) has been widely applied in the surgical system of large hospitals, including surgical training, simulation, and surgical planning. Both in medical education and surgical practice optimization, it has achieved significant advantages over traditional methods (6–8). A systematic review confirmed that 3D-printed organ models can significantly improve trainees’ anatomical understanding and surgical planning capabilities (8), while 3D printing simulators have been validated for their effectiveness in enhancing surgical skill acquisition (7). However, due to the complex anatomical structures of internal organs and high costs in the past, its application in undergraduate clinical internship teaching in developing countries is rarely reported. With the rapid popularization of 3D printing in provincial hospitals and the significant reduction in equipment and material costs, 3D Intelligent Printing Technology (3DIPT) has become increasingly accessible for clinical education. Constructivist learning theory holds that learning is an active process of knowledge construction by learners through hands-on practice and interactive exploration, rather than passive acceptance of information. In this study, 3DIPT-assisted teaching constructs a tangible learning scene for interns through organ model printing, interactive discussion and cross-disciplinary practice, which enables interns to actively explore anatomical knowledge and clinical application methods, and realize the construction and internalization of professional knowledge. This study aims to explore the impact of 3DIPT-assisted teaching on improving core competencies of clinical medicine interns, filling the research gap in context-adaptive medical education for developing countries and providing empirical evidence for the application of constructivist learning theory.

### Research objectives

Core competencies in this study refer to the integrated clinical and learning abilities required for clinical medicine interns to adapt to postgraduate further education and community health service practice, including five core dimensions: medical learning interest, self-directed anatomy learning ability, mobile learning willingness, transformational learning readiness, and National Medical Licensing Examination (NMLE)-related anatomical imaging application proficiency, which are consistent with the national clinical medicine undergraduate training standards in China.

This study focuses on five core dimensions of intern competencies: medical interest, self-directed anatomy learning ability, mobile learning willingness, transformational learning ability, and NMLE-related anatomical imaging proficiency. The specific objectives are: (1) To compare the differences in core competencies between interns receiving 3DIPT-assisted teaching and traditional teaching; (2) To verify the effectiveness of 3DIPT in addressing learning motivation deficits and enhancing clinical practice capabilities; (3) To propose a feasible teaching model balancing postgraduate education and community employment needs for developing countries.

### Significance of the study

Theoretically, this study is the first to integrate 3DIPT into the clinical internship education system, expanding the application scope of constructivist learning theory in undergraduate medical education in developing countries. Practically, the research results provide an operable intervention plan for medical educators, helping to improve the quality of clinical internship teaching, enhance interns’ competitiveness in postgraduate examinations and NMLE, and ultimately promote the development of primary healthcare services in developing countries.

## Materials and methods

### Study design and participants

This quasi-randomized controlled trial was conducted at a provincial hospital in China from February 2025 to August 2025. A total of 132 clinical medicine interns were enrolled, with sample size calculated using SPSS 23.0 (*α* = 0.05, *β* = 0.2, standard mean difference = 3.5, mean difference = 2.1), and expanded by 10% to account for potential attrition and loss to follow-up during the study. Participants were randomly assigned to the control group (even random numbers) or observation group (odd random numbers) at a 1:1 ratio (n = 66 per group). During the research, 12 interns were excluded (1 with interrupted internship due to special circumstances, 5 with unqualified internship attendance, 6 with incomplete research data). Finally, 120 eligible interns were included in the final statistical analysis (60 per group). Outcome assessments were performed by researchers blinded to group allocation to minimize potential bias.

### Inclusion criteria

(1) Aged ≥22 years; (2) Completed undergraduate theoretical courses and in the final year of clinical internship; (3) No mental illness or psychological abnormalities; (4) Understood the study purpose and volunteered to participate.

### Exclusion criteria

(1) Interrupted internship due to special circumstances; (2) Consecutive leave ≥3 days or cumulative leave ≥7 days during the internship; (3) Participated in other teaching method training; (4) Incomplete data.

### Teaching interventions

#### Control group

Interns received traditional internship teaching based on the standard Clinical Medicine Internship Manual, covering surgical and imaging system (radiology and ultrasound departments) rotations for 6 months. Teaching methods included bedside teaching, case discussions, and theoretical lectures.

#### Observation group

**Figure 1:**
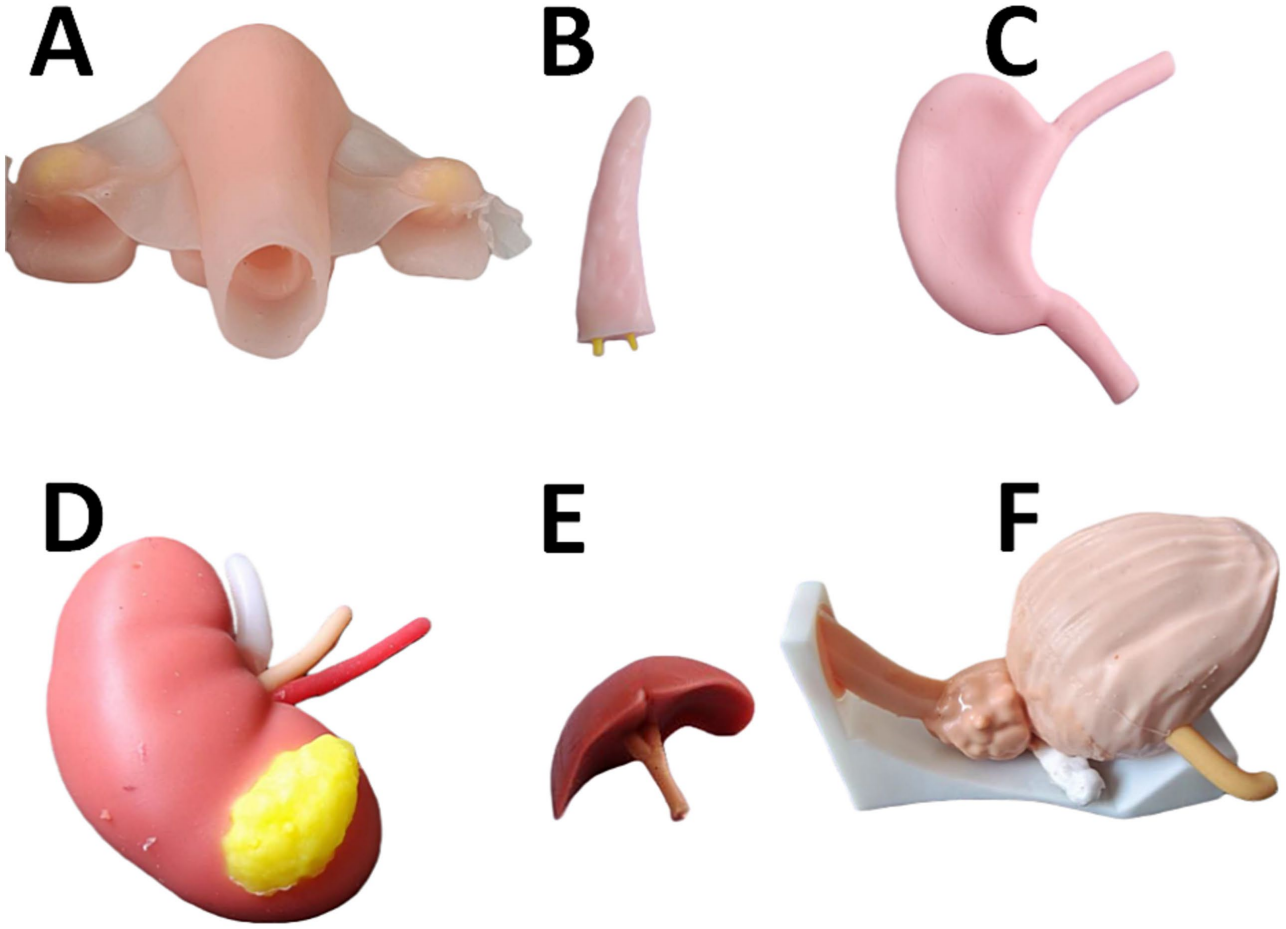
3D-printed organ models used for teaching: **(A)** Uterus + ovaries; **(B)** Pancreas; **(C)** Stomach; **(D)** Kidney (including arteries, veins, and ureters); **(E)** Bile duct; **(F)** Bladder + prostate (including vas deferens).

In addition to conventional teaching, the observation group received 3DIPT-assisted teaching for 6 months, with the following implementation steps:

1. Platform Construction: The teaching team and interns jointly selected core surgical organs. Anatomical and imaging textbooks/literatures were reviewed, and hospital MDCT image data were retrieved for 3D reconstruction using Zhongnan e3D Digital Medical Virtual Software V17.06 (China).
2. Model Printing: Ultimaker Cura 4.4.1 open-source slicing software (USA) was used to analyze lesions and ducts, generate G-code, and print organ models with anatomical layers and lesion features using Zhongrui Zhichuang SL600 stereolithography equipment (China) with composite materials of soft and hard resins. Models were dyed for teaching (Figure 1). The 3D reconstruction and slicing files of organ models were independently designed and produced by our research team based on clinical MDCT data of the hospital, and the self-developed G-code files were used for localized printing without universal shared files. The model production process was customized according to the teaching needs of clinical internship and the characteristics of NMLE examination points.
3. Interactive Discussion: For complex organs, 15–20 min WeChat group video conferences were held, involving rotating department teachers, 3D printing professionals, and interns to analyze anatomical structures, model printing quality, and clinical practice key points. Notably, 3D printing simulators have been widely validated for their effectiveness in surgical training, providing tangible and realistic models that enhance skill acquisition (7). A systematic review further confirmed that 3D-printed organ models, such as virtual kidney models for renal cancer surgery, significantly improve trainees’ anatomical understanding and surgical planning capabilities (8).

### Outcome measures

#### Primary outcome

Simulated NMLE anatomical imaging test: A 100-point test was developed by the research team based on 1999–2025 NMLE anatomical imaging questions, reflecting interns’ ability to apply theoretical knowledge to clinical examinations. This test is critical for both postgraduate students and community physicians, as it verifies core clinical competencies.

#### Secondary outcomes

##### Social medical curiosity (SMC) score

Assessed using the localized and validated Medical Curiosity Scale (9), focusing on the SMC dimension (Cronbach’s *α* = 0.88) to evaluate medical interest. The scale includes 2 dimensions and 10 items, with the Intellectual Medical Curiosity (IMC) dimension excluded due to insignificant changes during learning (*p* > 0.05) (10).

##### Self-directed anatomy learning ability score

Measured by the Self-directed Learning Scale for Medical Students’ Human Anatomy (11) (Cronbach’s *α* = 0.8–0.9), including learning motivation and learning strategy subscales. This scale was used due to the lack of specialized imaging self-directed learning scales.

##### Mobile learning willingness scale for medical students online (MLWS-MS) score

A validated scale with Cronbach’s *α* = 0.953, assessing interns’ willingness and ability to conduct mobile learning (12), which is crucial for fragmented learning in clinical settings.

##### Medical student transformational learning readiness scale (MSTLR) score

A scale with Cronbach’s *α* = 0.91, evaluating interns’ ability to integrate medical knowledge with 3D printing technology (an engineering discipline) and cross-disciplinary problem-solving skills (13).

### Data collection and statistical analysis

Data were collected via questionnaires, self-assessment reports, and knowledge tests before and after the 6-month intervention. All data were analyzed using SPSS 29.0 (SPSS Inc., Chicago, IL, USA). Categorical variables were presented as *n* (%) and compared using Pearson’s chi-square test or Fisher’s exact test (for sample size < 40 or theoretical frequency T < 1). Continuous variables were tested for normality using the Shapiro–Wilk test; normally distributed data were presented as mean ± standard deviation and compared using independent samples t-test, while non-normally distributed data were presented as median (25th percentile, 75th percentile) and compared using the Wilcoxon rank-sum test. Descriptive statistics for all continuous outcome variables were presented with mean ± standard deviation to reflect data distribution characteristics. Between-group differences in primary and secondary outcomes were analyzed by independent samples t-test, which is a commonly used statistical method for clinical education intervention studies with homogeneous baseline characteristics of participants. Statistical significance was set at *p* < 0.05.

### Ethical approval

This study was approved by the Medical Ethics Committee of Hangzhou Medical College (Approval No.: LL2025-011). All procedures performed in this study involving human participants were in accordance with the Declaration of Helsinki and its later amendments or comparable ethical standards. All participants provided written informed consent, and data were collected anonymously to protect privacy. No patients were involved, and there was no risk of physical or psychological harm.

## Results

### Baseline characteristics

There were no significant differences in baseline characteristics (age, gender, ethnicity, only-child status, GPA) between the two groups (all *p* > 0.05), indicating comparable baseline conditions (Table 1).

**Table 1:** Baseline characteristics of participants in the control and observation groups.

Parameters	Traditional teaching group (*n* = 60)	SFPC teaching group (*n* = 60)	Test statistic (*t*/*χ*^2^)	*p*-value
Age (years)	22.31 ± 0.94	22.47 ± 0.91	0.853	0.952
Gender (M/F)	15 (25.00%)/45(75.00%)	16 (26.67%)/44 (73.33%)	0.032	0.857
Ethnicity (Han)	58 (96.67%)	59 (98.33%)	0.338	0.561
Residence (Urban/Rural)	16 (26.67%)/44 (73.33%)	17 (28.33%)/43 (71.67%)	0.075	0.784
Only-child (Yes)	14 (23.33%)	15 (25.00%)	0.069	0.792
Undergraduate GPA	4.81 ± 0.73	4.75 ± 0.61	0.484	0.629

Continuous variables are presented as mean ± standard deviation and compared using independent samples *t*-test; categorical variables are presented as *n* (%) and compared using Pearson’s chi-square test.

### Comparison of outcome measures before and after intervention

#### Social medical curiosity (SMC) score

**Figure 2:**
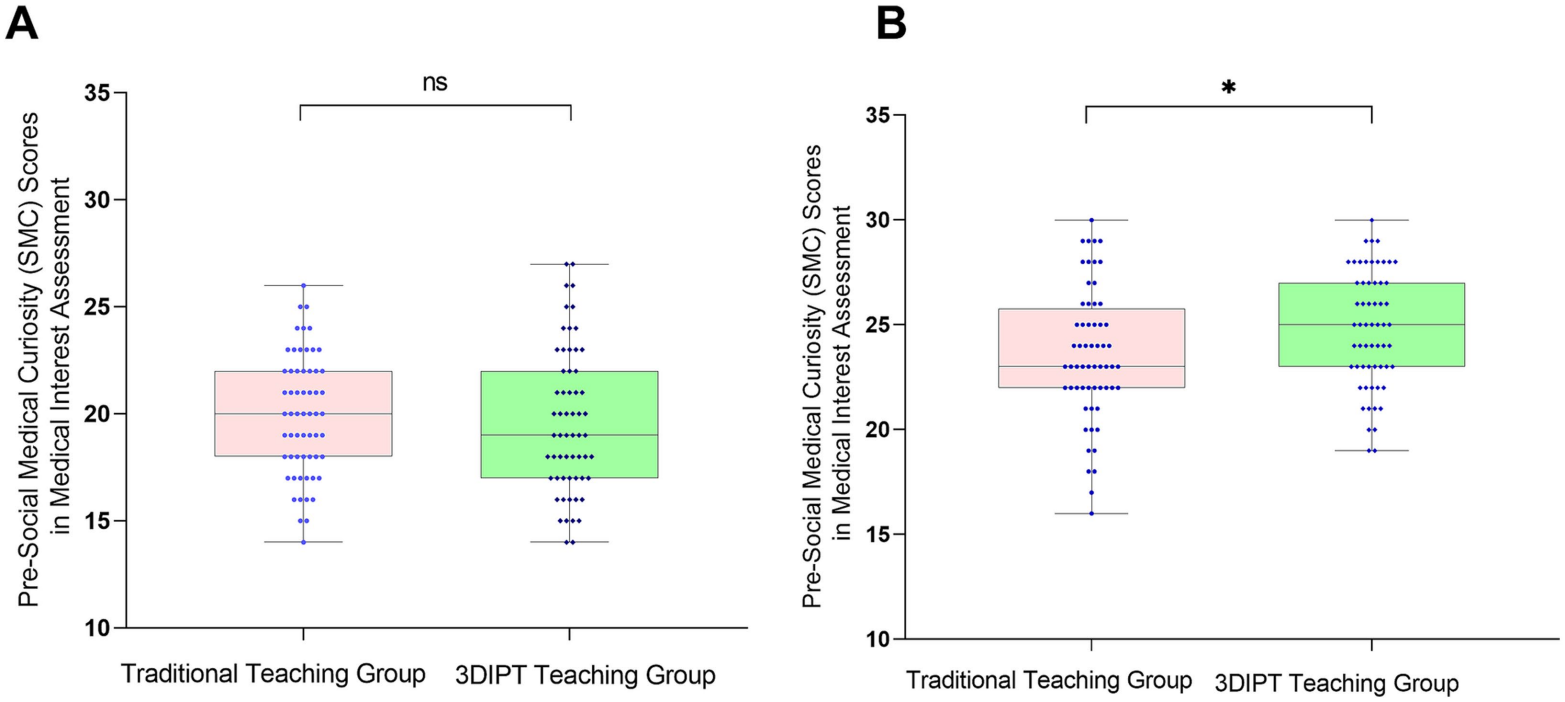
Comparison of SMC scores between the two groups before and after intervention. **(A)** Pre-intervention (baseline) status; **(B)** Post-intervention status. Data are presented as mean ± SD. *p < 0.05 vs. control group post-intervention.

No significant difference was observed between the two groups before intervention (19.65 ± 3.42 vs19.93 ± 3.42, t = 0.4999, *p* = 0.6181). After intervention, the observation group had a significantly higher SMC score than the control group (24.77 ± 2.76 vs23.57 ± 3.17, *t* = 2.215, *p* = 0.0287) (Figure 2), the between-group difference was statistically significant, indicating the potential effectiveness of 3DIPT-assisted teaching in improving the corresponding core competency of interns.

#### Self-directed anatomy learning ability score

**Figure 3:**
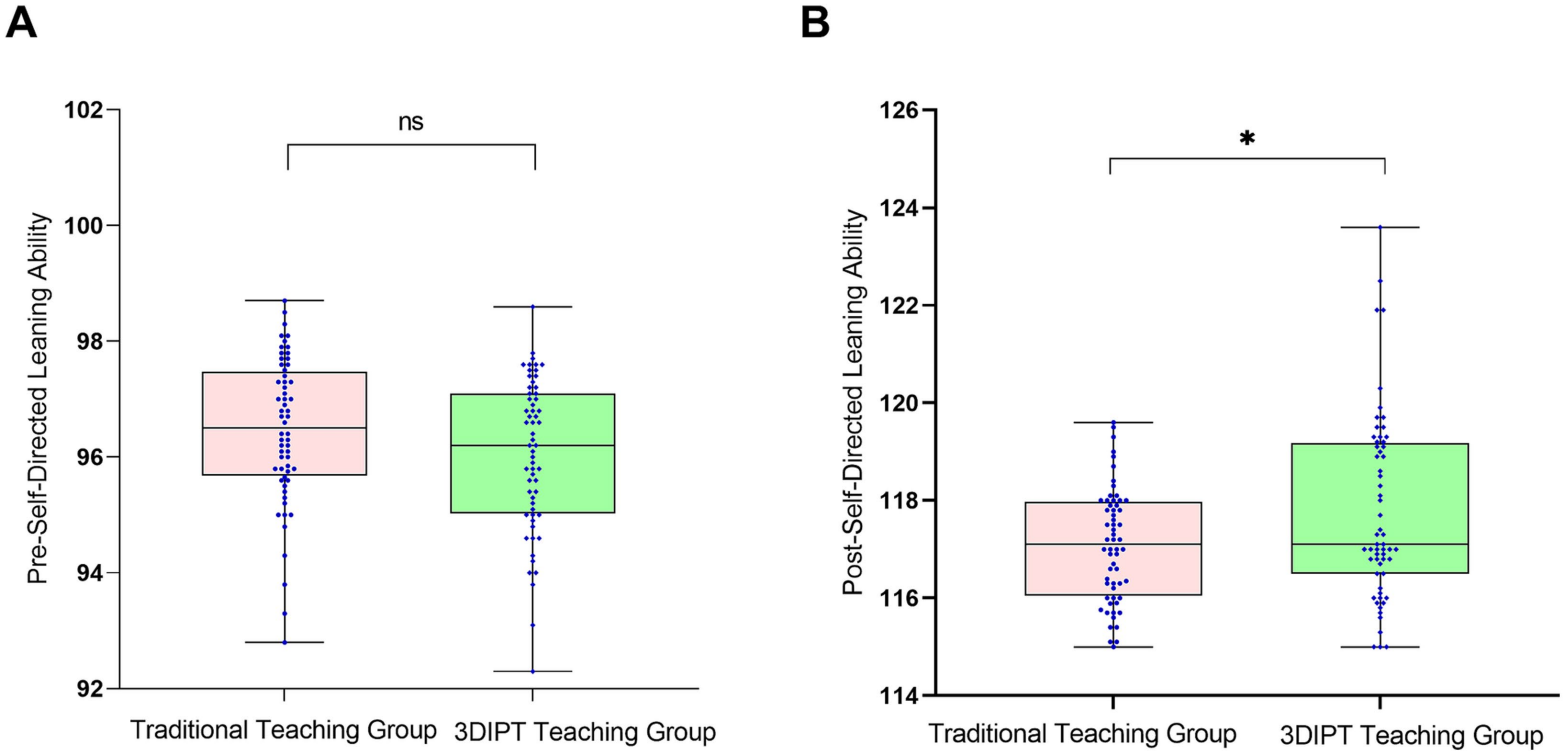
Comparison of self-directed anatomy learning ability scores between the two groups before and after intervention. **(A)** Pre-intervention (baseline) status; **(B)** Post-intervention status. Data are presented as mean ± SD. *p < 0.05 vs. control group post-intervention.

Pre-intervention scores were similar between groups (96.95 ± 2.55 vs. 96.45 ± 2.45, *t* = 1.323, *p* = 0.1883). Post-intervention, the observation group showed a significantly higher score (118.95 ± 3.15 vs. 117.10 ± 3.56, *t* = 2.087, *p* = 0.0391) (Figure 3), the between-group difference was statistically significant, indicating the potential effectiveness of 3DIPT-assisted teaching in improving the corresponding core competency of interns.

#### MLWS-MS score

**Figure 4:**
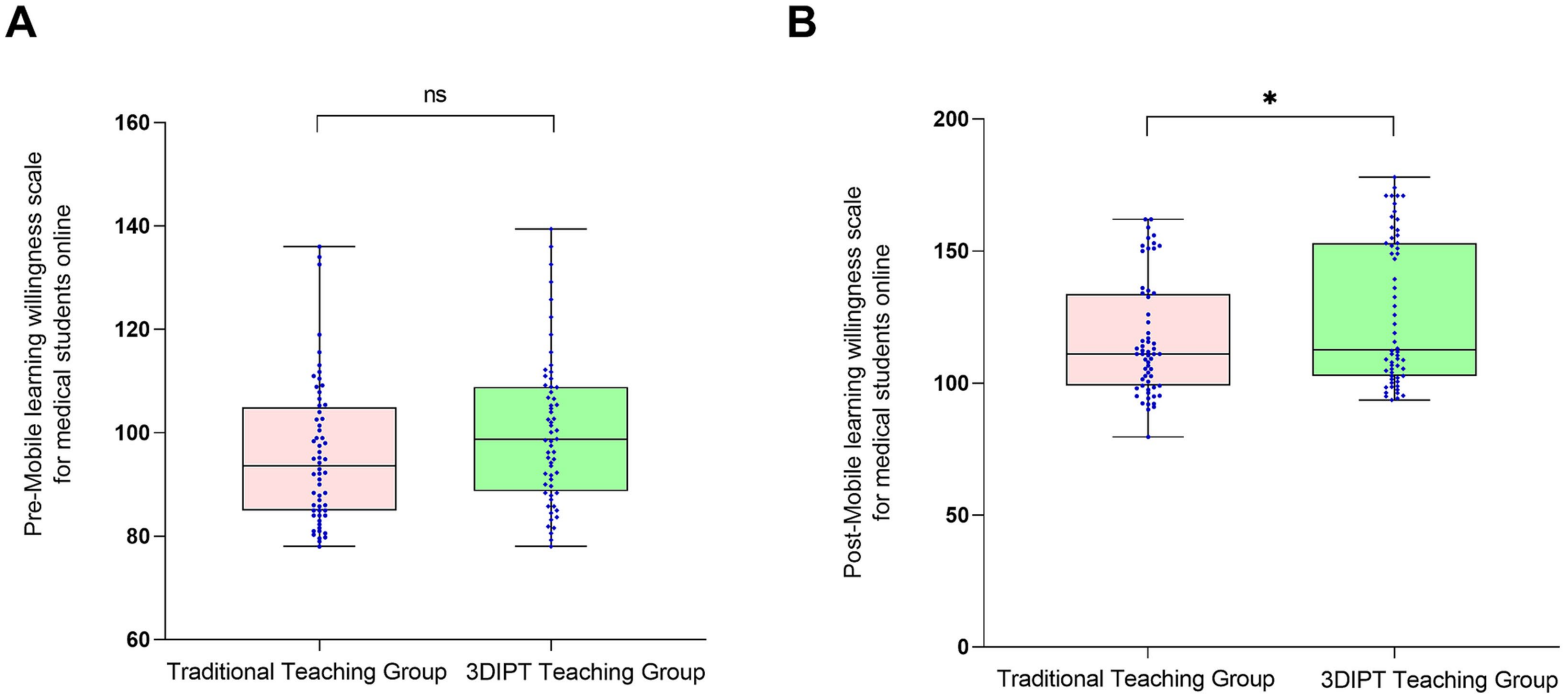
Comparison of MLWS-MS scores between the two groups before and after intervention. **(A)** Pre-intervention (baseline) status; **(B)** Post-intervention status. Data are presented as mean ± SD. *p < 0.05 vs. control group post-intervention.

No significant pre-intervention difference was found (95.90 ± 4.45 vs. 100.50 ± 4.57, *t* = 1.730, *p* = 0.0863). Post-intervention, the observation group had a significantly higher score (126.60 ± 10.35 vs. 116.40 ± 10.20, t = 2.242, *p* = 0.0268) (Figure 4), the between-group difference was statistically significant, indicating the potential effectiveness of 3DIPT-assisted teaching in improving the corresponding core competency of interns.

#### MSTLR score

**Figure 5:**
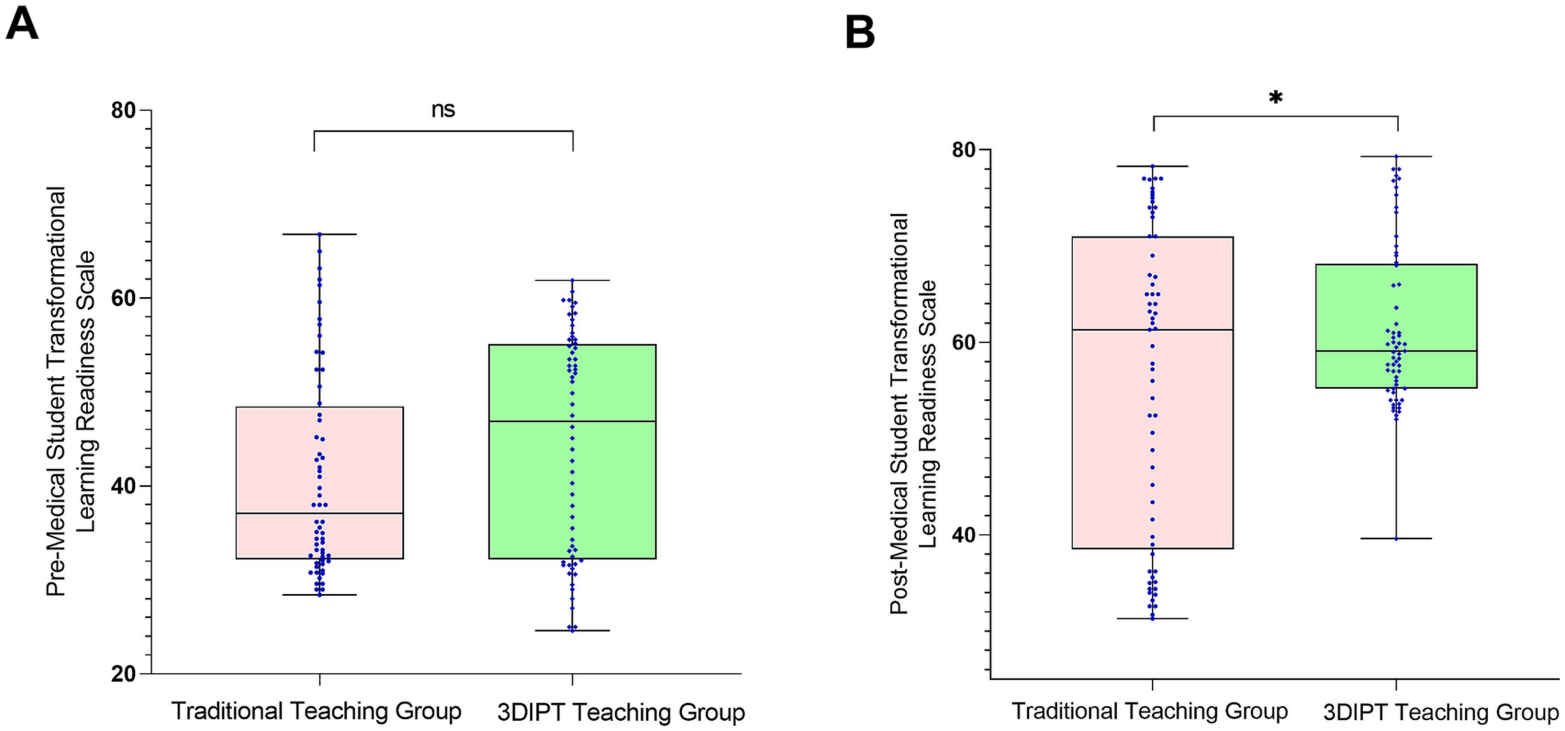
Comparison of MSTLR scores between the two groups before and after intervention. **(A)** Pre-intervention (baseline) status; **(B)** Post-intervention status. Data are presented as mean ± SD. *p < 0.05 vs. control group post-intervention.

Pre-intervention scores were comparable (41.05 ± 3.42 vs. 44.50 ± 3.60, *t* = 1.648, *p* = 0.1019). Post-intervention, the observation group showed a significantly higher score (61.50 ± 5.35 vs. 56.10 ± 5.20, t = 2.315, *p* = 0.0223) (Figure 5), the between-group difference was statistically significant, indicating the potential effectiveness of 3DIPT-assisted teaching in improving the corresponding core competency of interns.

#### Simulated NMLE anatomical imaging test score

**Figure 6:**
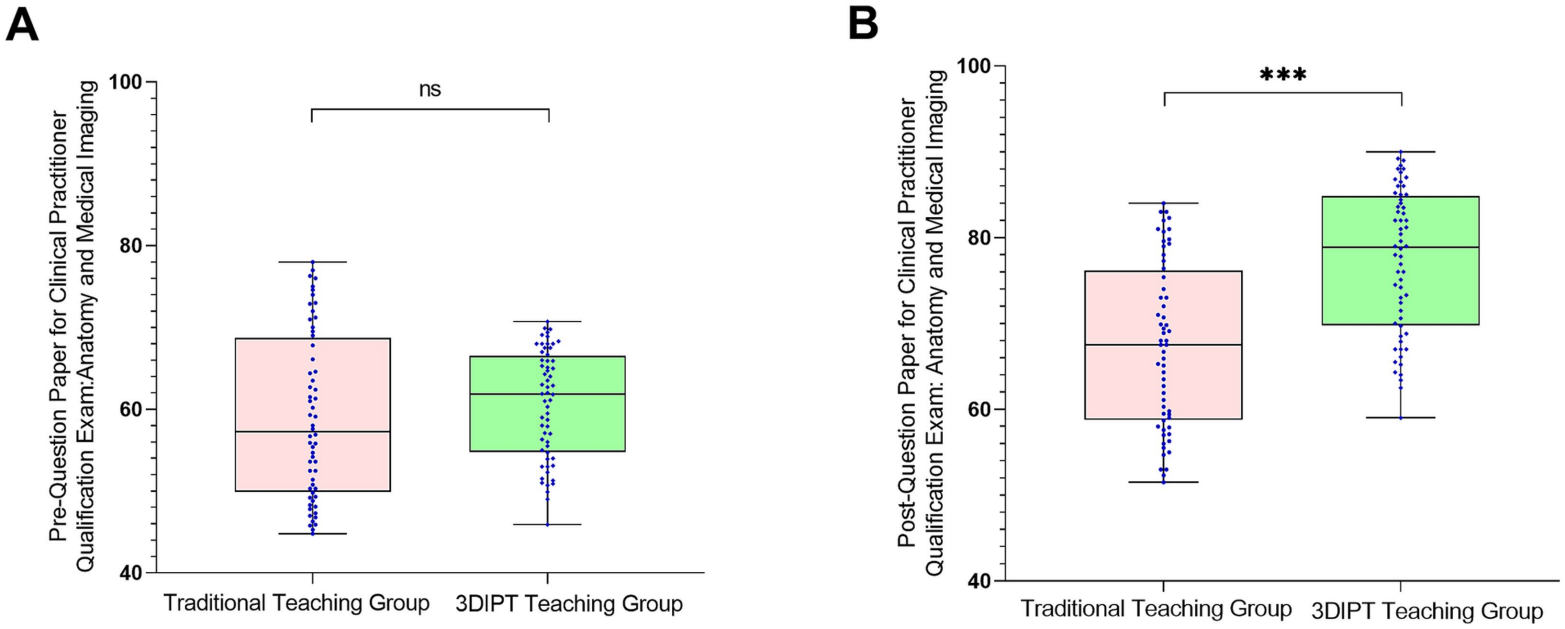
Comparison of simulated NMLE anatomical imaging test scores between the two groups before and after intervention. **(A)** Pre-intervention (baseline) status; **(B)** Post-intervention status. Data are presented as mean ± SD. ***p < 0.001 vs. control group post-intervention.

Pre-intervention scores were similar (59.10 ± 9.5 vs. 60.60 ± 8.8, *t* = 0.9983, *p* = 0.3202). Post-intervention, the observation group had a significantly higher score (77.30 ± 9.65 vs. 67.36 ± 9.55, *t* = 6.032, *p* < 0.001) (Figure 6), the between-group difference was statistically significant, indicating the potential effectiveness of 3DIPT-assisted teaching in improving the corresponding core competency of interns.

## Discussion

This study demonstrates that 3DIPT-assisted teaching significantly improves core competencies of clinical medicine interns in developing countries, with positive effects on medical interest, self-directed learning, mobile learning willingness, transformational learning ability, and NMLE performance. These findings align with the study’s core objective of addressing the dual pressures of postgraduate education and community employment for interns in developing countries.

### Mechanisms underlying the effectiveness of 3DIPT

#### Enhanced medical interest

The interactive nature of 3DIPT—where interns participate in organ model design, reconstruction, and printing—transforms abstract anatomical knowledge into tangible models, fostering exploratory interest in medicine (9). This aligns with Yang et al.’s (9) finding that hands-on application drives the conversion of external emotional motivation to internal professional interest, addressing the issue of insufficient learning motivation in traditional internship teaching.

### Improvement of learning attention deficit by alleviating cognitive resource occupation

Clinical medicine interns face dual pressures of postgraduate entrance examination and NMLE, whose cognitive resources are often overoccupied by exam-oriented rote learning, leading to learning attention deficit and reduced learning efficiency in clinical internship (14). 3DIPT-assisted teaching transforms abstract anatomical knowledge into tangible and operable model learning, which reduces the cognitive load of interns in understanding complex anatomical structures (15), effectively alleviates the occupation of limited cognitive resources, and thus improves the learning attention deficit in clinical education, making interns more focused on the integration of theoretical knowledge and clinical practice.

### Embodied cognition as the theoretical core of 3DIPT-assisted teaching

The effectiveness of 3DIPT-assisted teaching is essentially based on the core connotation of embodied cognition theory, which holds that cognitive activities are closely linked to physical body perception and interactive practice (16, 17). Interns not only obtain visual and auditory knowledge through traditional teaching, but also grasp the structural characteristics of human organs and the relationship between lesions and normal tissues through hands-on operation of 3D-printed models, realizing the integration of body perception and cognitive learning. This embodied learning mode conforms to the law of medical knowledge acquisition, and is an important embodiment of technology-enhanced learning in modern medical education, which can effectively promote the internalization and application of clinical knowledge.

### Promoted self-directed learning

3DIPT creates a “demand-driven” learning cycle: interns actively seek anatomical and imaging materials to solve model printing problems, enhancing autonomy and initiative (11). This positive feedback loop helps interns cope with the pressures of postgraduate examinations and NMLE, laying a solid foundation for community clinical practice.

### Strengthened mobile learning capabilities

The popularity of smartphones and tablets has made mobile learning a key component of clinical education (18). 3DIPT encourages interns to use mobile apps for knowledge retrieval and group discussions, improving their ability to integrate fragmented learning into daily practice (12). This is particularly important for community physicians who lack long-term advanced training opportunities in large hospitals.

### Cultivated transformational learning ability

3D printing, a cross-disciplinary technology combining medicine and engineering, provides a unique platform for transformational learning (13). Interns integrate medical knowledge with engineering technology, developing critical thinking and cross-disciplinary problem-solving skills—consistent with the goals of third-generation medical education reform (19). This addresses the gap between theoretical knowledge and clinical practice in developing countries.

### Improved NMLE performance

3DIPT shifts learning from rote memorization to application-oriented learning, facilitating deep knowledge internalization (6). The sense of achievement from successful model printing further motivates interns to invest in exam preparation, leading to improved test scores (9). This “motivation activation + competency training” dual-track model effectively supports both postgraduate studies and community employment.

### International application of 3DIPT and accessibility in rural areas

3DIPT has been widely applied in surgical training and medical education in Europe and America, with mature standardized model databases and cross-institutional sharing mechanisms. In China, 3DIPT is rapidly popularized in provincial and municipal hospitals, but its application in rural and grassroots medical institutions is limited by factors such as equipment cost and professional technical personnel. However, with the reduction of 3D printing material cost and the popularization of remote technical support, simplified 3DIPT teaching models are expected to be promoted in grassroots medical education.

### Theoretical and practical implications

Theoretically, this study provides new empirical evidence for constructivist learning theory in undergraduate medical education in developing countries, highlighting the role of 3DIPT in bridging the gap between theoretical knowledge and clinical practice (20, 21). Practically, the 3DIPT teaching model offers an operable solution for medical educators: hospitals and universities can establish 3D printing teaching platforms to enhance interns’ professional value recognition, improving the quality of clinical internship teaching (22–24) and promoting the development of primary healthcare services.

From the perspective of public health, the improvement of interns’ core competencies by 3DIPT-assisted teaching is conducive to strengthening the construction of primary health care talent team in developing countries, improving the clinical service ability of community physicians, and further promoting the balanced development of medical and health services between urban and rural areas. The teaching model can be further optimized according to the actual needs of community health services, and provide technical support for the training of grassroots medical talents with practical clinical abilities.

### Limitations

This study has several limitations: (1) It is a single-center study, limiting the generalizability of results to other medical institutions in developing countries; (2) Subgroup analysis based on gender, educational background, or career intentions was not performed due to sample size constraints; (3) The 6-month intervention period only reflects short-term effects, and long-term follow-up is needed to evaluate sustained competency improvement; (4) Self-reported scales may introduce response bias, and may also lead to social desirability bias and Hawthorne effect, and there may be potential information contamination between the two groups during the internship teaching process; future studies should integrate qualitative methods such as interviews and clinical observations for comprehensive analysis; (5) As this study is the first comprehensive application of 3DIPT in the teaching of core organ systems for clinical medicine interns with learning interests and knowledge deficiencies, the research team previously only applied 3D printing in the single disease teaching of liver cancer. In this study, the core energy of the team was focused on the collection of multi-detector computed tomography imaging data, the optimization of 3D modeling and printing of core organ systems, and the design and implementation of teaching schemes. Thus, the investment in the refined collection of interns’ baseline data was insufficient, and some baseline data dimensions were not completely collected. This leads to the failure of baseline correction analysis for outcome indicators, the lack of reporting of effect sizes and 95% confidence intervals, and the absence of multiple comparison correction. The above statistical limitations may cause potential bias in effect estimation and increase the risk of Type I error, which is a major shortcoming of this exploratory study; (6) This study only evaluated the short-term effect of 6-month intervention, and did not assess the long-term clinical performance and practice ability of interns after employment, which will be the focus of follow-up longitudinal research.

## Conclusion

3DIPT-assisted teaching may significantly improve core competencies of clinical medicine interns in developing countries, including medical interest, self-directed learning ability, mobile learning willingness, transformational learning ability, and NMLE performance. It may effectively address learning motivation deficits and provide a dual-track strategy balancing postgraduate education and community employment. As clinical medical education shifts toward competency-based training, 3DIPT holds great potential for promoting high-quality talent cultivation and improving clinical service levels in developing countries, a perspective further supported by a regional review highlighting the transformative value of 3D printing in advancing medical education and training in resource-limited settings (20). In subsequent research, on the basis that 3D printing technology and teaching programs have been mature, we will focus on improving the comprehensive collection of interns’ baseline data, and cooperate with professional statisticians in the team to optimize the statistical analysis scheme, adopt baseline correction analysis methods to reduce the bias of effect estimation, supplement the complete report of effect size and 95% confidence interval to reflect the practical value of intervention effect, and implement appropriate multiple comparison correction to ensure statistical rigor; at the same time, optimize the randomization and allocation concealment scheme, strictly control selection bias and confounding factors, and carry out multi-center, large-sample longitudinal studies to evaluate the impact of 3DIPT-assisted teaching on the long-term clinical practice ability of interns, so as to provide more robust empirical evidence for the promotion of this teaching model in the training of primary health care talents.

## Data Availability

The raw data supporting the conclusions of this article will be made available by the authors, without undue reservation.
